# Lewy pathology accumulates in enlarged corticostriatal synapses in α-synucleinopathies

**DOI:** 10.1093/braincomms/fcag409

**Published:** 2026-10-25

**Authors:** Alice Prigent, Aastha Singh, Michael X Henderson

**Affiliations:** Department of Neurodegenerative Science, Van Andel Institute, Grand Rapids, MI 49503, USA; Department of Neurodegenerative Science, Van Andel Institute, Grand Rapids, MI 49503, USA; Department of Neurodegenerative Science, Van Andel Institute, Grand Rapids, MI 49503, USA

**Keywords:** putamen, synapses, VGLUT1, Parkinson’s disease, dementia

## Abstract

Lewy body diseases, including Parkinson’s disease, Parkinson’s disease dementia and dementia with Lewy bodies, are neurodegenerative disorders characterized by the accumulation of misfolded α-synuclein in the form of Lewy pathology. A hallmark of these diseases is degeneration of the nigrostriatal pathway, resulting in loss of dopaminergic input to the striatum and consequent motor dysfunction. Lewy pathology is present in many regions outside the substantia nigra, and cortical Lewy pathology is the best correlate of cognitive decline in individuals who develop dementia. In addition, there is a high burden of neuritic Lewy pathology in the putamen, though the neuronal origin of this pathology is unclear. In the current study, we quantified the burden of Lewy pathology in the putamen across the spectrum of Lewy body diseases. Immunohistochemistry was used to quantify the Lewy burden in the putamen, cingulate and frontal cortices of 8 controls and 28 Lewy body disease cases. Even in Parkinson’s disease cases without dementia, we observed a nearly complete striatal dopaminergic denervation among Lewy body disease cases. Consistent with this denervation, most α-synuclein pathology did not colocalize with dopaminergic terminals, but was instead enriched in excitatory terminals. This enrichment in glutamatergic terminals was associated with swollen axons, but not with overall loss of those terminals. These findings suggest that Lewy pathology accumulates at excitatory corticostriatal synapses prior to overt synaptic degeneration and could contribute to cognitive decline in Lewy body diseases.

## Teaser

Prigent *et al*. report that Lewy pathology in Parkinson’s disease preferentially accumulates in excitatory corticostriatal synapses late in disease. These affected synapses are enlarged but not lost, suggesting that synaptic dysfunction precedes degeneration and may contribute to disease progression and cognitive impairment.

## Graphical Abstract

**Graphical Abstract:**
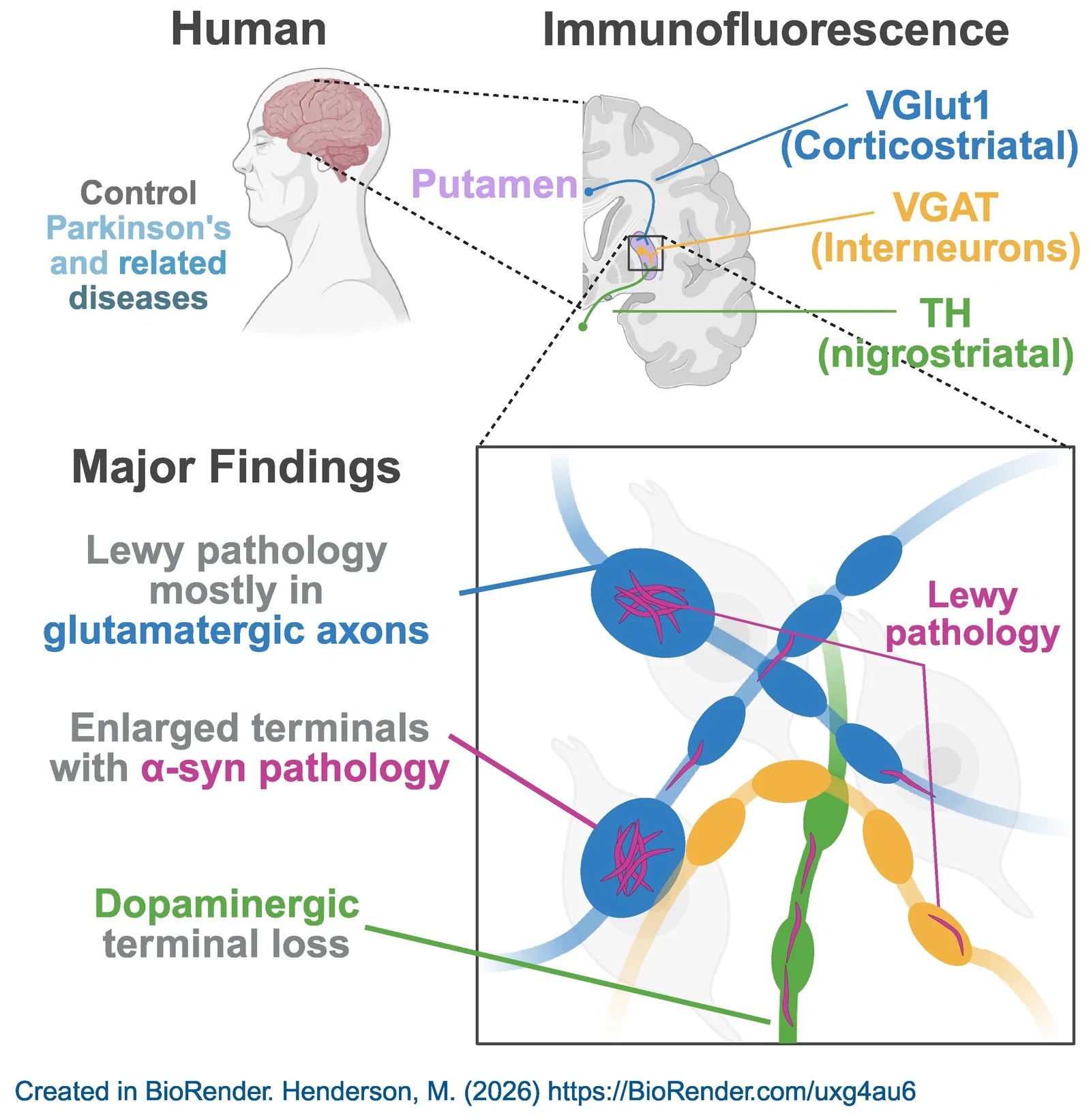

## Introduction

Parkinson’s disease (PD) is a progressive neurodegenerative disorder characterized primarily by motor symptoms such as bradykinesia, rigidity, tremor and postural instability, but also by a broad spectrum of non-motor symptoms, including autonomic dysfunction, sleep disturbances and cognitive impairment.^1-4^ Central to PD and related disorders is the accumulation of misfolded α-synuclein protein aggregates, known as Lewy pathology, in vulnerable brain regions.^5,6^ Over time, up to 80% of people with PD develop cognitive decline sufficient to warrant a diagnosis of Parkinson’s disease dementia (PDD).^7^ Dementia with Lewy bodies (DLB) shares overlapping clinical and pathological features with PDD,^8,9^ but is distinguished primarily by the timing of symptom onset: in DLB, cognitive symptoms emerge before or within one year of motor symptoms, whereas in PDD, dementia develops after a longer duration of established motor disease.^10-12^

Lewy pathology was initially staged in 2003 by Braak and colleagues, who identified a characteristic spatiotemporal progression initially emerging in the olfactory structures and lower brainstem before ascending through midbrain regions such as the substantia nigra pars compacta (SNpc) and eventually involving limbic and neocortical areas.^13^ However, in 2009, Beach and colleagues proposed the unified staging system for Lewy body diseases (LBDs) to better categorize cases where Lewy pathology is limited to the olfactory bulb or follows a limbic-predominant pathway that initially does not involve the brainstem.^14^ This system classifies Lewy pathology stages as: (i) olfactory bulb only, (ii) brainstem predominant or limbic predominant, (iii) brainstem and limbic and finally (iv) neocortical. This anatomical trajectory correlates broadly with the clinical trajectory of the disease: early non-motor symptoms, including hyposmia and autonomic dysfunction, correspond to pathology in peripheral and lower brainstem regions, while dopaminergic neuronal loss in the SNpc underlies the emergence of cardinal motor features.^3,14-16^ In later stages, the involvement of neocortical regions is associated with cognitive decline and neuropsychiatric symptoms,^17^ highlighting the multifaceted clinical impact of progressive α-synuclein aggregation throughout the nervous system.

Degeneration of SNpc neurons leads to loss of dopaminergic input to the putamen, a key structure within the basal ganglia.^18,19^ This loss of dopamine disrupts basal ganglia circuitry, resulting in impaired motor control.^20-22^ The putamen plays a crucial role not only in motor learning and execution but also in cognitive functions.^23^ It receives dense dopaminergic projections from the SNpc, glutamatergic inputs from cortical regions and the thalamus and GABAergic inputs from local collaterals as well as projections from globus pallidus externa.^24-26^ In addition to the loss of dopaminergic tone in the putamen, this region also accumulates abundant Lewy neurites.^27^ Interestingly, the burden of Lewy pathology in the putamen is even greater in PDD, DLB and Alzheimer’s disease than in PD alone, suggesting a relation of this pathology to dementia.^28-30^ However, the precise origin of Lewy neurites in the putamen and their direct contribution to symptoms such as dementia remain unclear.

Presynaptic enrichment of Lewy pathology and synaptic dysfunction are hypothesized to be an early phenomena in PD.^31-33^ Recent studies have shown that α-synuclein aggregates can accumulate at dopaminergic synapses in the putamen prior to terminal loss, supporting a synaptic origin for pathology progression.^34^ These proteinase K-resistant aggregates are predominantly present as small aggregates and colocalize with presynaptic markers such as synapsin I and synaptophysin.^35,36^ The presynaptic localization of Lewy pathology is associated with structural alterations such as dendritic spine degeneration in medium spiny neurons and frontal cortical neurons in DLB cases^36,37^ and with the formation of larger dopamine transporter (DAT)/α-synuclein complexes in the putamen of people with PD.^38^ However, it remains unclear whether Lewy pathology similarly affects other striatal afferents, particularly glutamatergic and GABAergic inputs, or how such changes relate to cortical Lewy burden in LBDs.

In the current study, we sought to determine which specific presynaptic inputs to the putamen—corticostriatal glutamatergic (VGLUT1-positive) or GABAergic (VGAT-positive)—are preferentially affected by Lewy pathology across PD, PDD and DLB. Additionally, we investigated whether α-synuclein accumulation at these terminals is associated with structural changes, such as synaptic enlargement, which may contribute to synaptic dysfunction. We examined the burden of cortical and striatal Lewy pathology in 8 controls, 9 idiopathic PD, 10 idiopathic PDD and 9 idiopathic DLB. Co-immunofluorescence was used to assess Lewy pathology colocalization with dopaminergic (TH; tyrosine hydroxylase), cortical glutamatergic (VGLUT1; vesicular glutamate transporter 1) and GABAergic (VGAT; vesicular GABA transporter) terminals in the putamen across all groups. All disease groups showed a nearly complete denervation of dopaminergic terminals, so only a minimal proportion of Lewy pathology was located in these terminals. Inhibitory VGAT-positive terminals were also only rarely associated with Lewy pathology. In contrast, the majority of Lewy pathology was associated with excitatory VGLUT1-positive terminals, which primarily arise from corticostriatal projections. Indeed, cortical Lewy pathology load was positively associated with putamen Lewy pathology. Interestingly, across LBDs, enlarged VGLUT1 puncta were observed, which colocalized with Lewy aggregates. Despite the enrichment of Lewy pathology in VGLUT1-positive terminals, there was no overall reduction in the number of terminals. Taken together, our results suggest that Lewy pathology accumulation at cortical excitatory synapses may precede detectable synaptic degeneration in the putamen and could contribute to the cortical propagation of Lewy pathology and progression to cognitive decline.

## Materials and methods

### Human brain tissue

A total of 36 brains were used in this study: 8 controls, 9 PD, 10 PDD and 9 DLB. All brain tissues were obtained from the Center for Neurodegenerative Disease Research (CNDR) at the University of Pennsylvania. The research conducted on human tissue was approved as non-human subjects research. Frozen tissue was briefly thawed and transferred to 10% neutral buffered formalin (NBF) for overnight fixation. The fixed tissue was embedded in paraffin after 24 h for further histological examination. Primary pathology to estimate % area occupied by pS129 α-synuclein was conducted on these tissues as previously published.^30^ pS129 α-synuclein is used as a pathological marker in human cases due to its high sensitivity and absence of positivity in control cases.

### Immunofluorescence

Slides were de-paraffinized with two sequential 5 min washes in xylenes, followed by 1 min washes in a descending series of ethanols: 100%, 100%, 95%, 80%, 70%. Slides were then incubated in deionized water for 1 min and then transferred to the BioGenex EZ-Retriever System, where they were incubated in antigen unmasking solution (Vector Laboratories; Cat #H3300) and microwaved for 15 min at 95°C. Slides were allowed to cool for 20 min at room temperature, washed in running tap water for 10 min and then blocked for 1 h at room temperature in 0.1 M Tris/2% foetal bovine serum (FBS). Primary antibodies were incubated in 0.1 M Tris/2% FBS in a humidified chamber overnight at 4°C. The following antibodies were used: mouse monoclonal anti-pS129 α-synuclein (81A) (1:1000; Biolegend, Cat #MMS-5091, RRID:AB_2564891), guinea pig anti-VGLUT1 (1:1000; Synaptic Systems, Cat #135 138, RRID:AB_887875), rabbit polyclonal anti-VGAT (1:1000; Synaptic Systems, Cat #131 002, RRID:AB_887871) and mouse monoclonal anti-TH (1:1000; Sigma-Aldrich, Cat #T2928, RRID:AB_477569). Primary antibodies were rinsed off with 0.1 M Tris for 5 min and then incubated with the appropriate secondary antibody for 1 h at room temperature: Goat anti-Rabbit IgG (H + L), Alexa Fluor™ 488 (1:1000; Thermo Fisher Scientific, Cat #A-11008; RRID:AB_143165), Goat anti-Guinea Pig IgG (H + L), Alexa Fluor™ 568 (1:1000; Thermo Fisher Scientific, Cat #A-11075; RRID:AB_2534119), Goat anti-Mouse IgG1, Alexa Fluor™ 568 (1:1000; Thermo Fisher Scientific, Cat #A-21124; RRID:AB_2535766), Goat anti-Mouse IgG2a, Alexa Fluor™ 647 (1:1000; Thermo Fisher Scientific, Cat #A-21241; RRID:AB_2535810). Secondary antibodies were rinsed off with 0.1 M Tris for 5 min and then treated with Sudan black B (Thermo Fisher Scientific, Cat #190160250) for 45 s. Slides were rinsed off and then mounted with coverglass in ProLong^TM^ Gold with DAPI (Invitrogen, Cat #P36931).

Confocal 2D images were collected sequentially with a Galvano scanner on a Nikon A1plus-RSi equipped with an Eclipse Ti2-E inverted base and acquired with Nikon Elements (version 5.21) software using 405, 488, 561 and 633 nm solid state laser lines. Emitted light was detected through a Nikon Plan Apochromat VC 60x/1.4NA oil immersion objective, using a DU4 multi-alkali PMT detector. To increase the size of the imaged regions, large images were generated by stitching 1024 × 1024 pixel tiles, with 5% overlap. For TH analysis, three regions with a final stitched size of 3943 × 3943 pixels (corresponding to an area of 808 μm × 808 μm) each were imaged from the putamen of the donors. For the synaptic markers, four images with a stitched image size of 1997 × 1997 pixels (corresponding to an area of 409.2 μm × 409.2 μm) each were obtained. All images were captured with a scan speed of 0.25 frames/sec, optical zoom of 2× and a confocal pinhole aperture of 89.4 μm.

### Object-based colocalization and object size quantification

Fluorescence images were imported into CellProfiler software (CellProfiler v4.2.6). After applying the RescaleIntensity module, primary objects were detected using the speckles (synaptic markers) and neurites (pSyn) features. Synaptic objects with diameters < 0.4 μm and pSyn objects < 1 μm were excluded from analysis. Child and parent objects were assigned and the parent with the most overlap was considered colocalized with the child object. pSyn objects colocalizing with VGLUT1-positive and VGAT-positive terminals were defined as pSyn/VGLUT1+ and pSyn/VGAT+, respectively. The total number of colocalized objects and object size (µm^2^) were then determined.

For TH analysis, primary objects were detected using the neurite feature. pSyn objects colocalizing with TH are defined as pSyn/TH+. Area occupied by TH and percentage of pSyn colocalized objects were then determined.

### Statistical analysis

The statistical analyses were performed in GraphPad Prism software version 9.3.1 (GraphPad Software Inc., La Jolla, CA, USA). The data shown in this study are mean ± standard error of the mean (SEM). Normality of the data was assessed using the D'Agostino–Pearson test and the Shapiro–Wilk test. Comparisons between two groups were performed using Welch’s unpaired *t*-test. For comparison among multiple groups, Welch’s one-way ANOVA followed by Dunnett’s T3 multiple comparisons test was used when assumptions of normality or equal variances were not met (specified in the figure legend). When the data satisfied assumptions of normality and homogeneity of variance, one-way ANOVA followed by Tukey’s multiple comparisons test was applied. Linear regressions and correlation coefficients were calculated in GraphPad Prism.

## Results

### Case demographics

A total of 36 cases were included in this study: 8 controls (CTRL), 9 Parkinson’s disease (PD), 10 Parkinson’s disease with dementia (PDD) and 9 DLB. The sex, postmortem interval, age of death, disease duration and Braak stage for each case are listed in Supplementary Table S1. Despite efforts to match for age, control cases were significantly younger than the PD donors (61 versus 75 years, *P* = 0.003) and the PDD donors (61 versus 71 years, *P* = 0.034). No significant difference was observed between the control and DLB donors (61 versus 71 years, *P* = 0.052) (Fig. 1A). Disease duration was significantly shorter in DLB cases compared to PD and PDD cases (Fig. 1B). The postmortem interval, representing the time elapsed between death and freezing, was similar across groups (Fig. 1C). For most of the cohorts, there were more males than females (CTRL: *F* = 37.5%; PDD: *F* = 10%; DLB: *F* = 11.1%) except the PD group (*F* = 55.6%, Fig. 1D).

**Figure 1:**
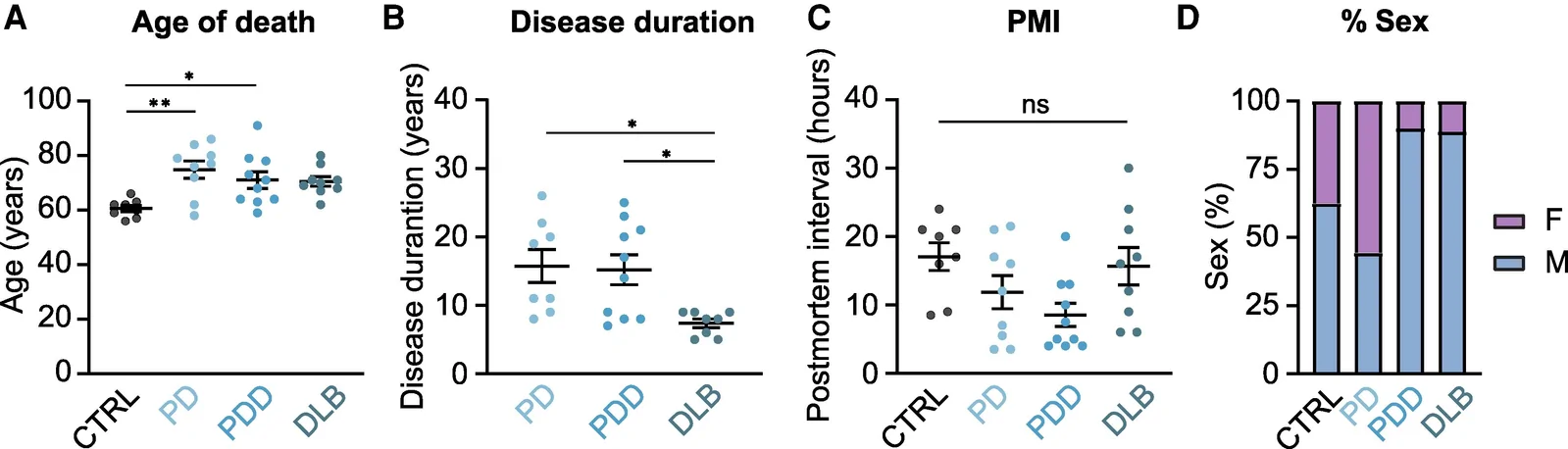
**Demographic and clinical characteristics of study cohort.** (**A**) Age at death for control (CTRL, *n* = 8), Parkinson’s disease (PD, *n* = 9), PD dementia (PDD, *n* = 10) and dementia with Lewy bodies (DLB, *n* = 9) (*F*(3,32) = 5.330, *P* = 0.004). (**B**) Disease duration (*F*(2,23) = 5.385, *P* = 0.012). (**C**) Post-mortem interval (PMI) (*F*(3,32) = 2.955, *P* = 0.047). (**D**) Percentages of the total group represented by each sex. Females (F) are shown in violet and males (M) in blue. Error bars represent mean ± SEM with data from individual cases plotted as dots. One-way ANOVA; Tukey’s multiple comparisons test. *P < 0.05, **P < 0.01, ns = non-significant.

### Striatal Lewy pathology burden is associated with cortical Lewy pathology across α-synucleinopathies

The burden of α-synuclein pathology in the putamen is not well-characterized and is not included in general staging systems.^13,14^ However, in a previous study focused on the relationship between lipid metabolites and protein pathologies, we observed abundant Lewy neurites in the putamen of PD, PDD and DLB cases.^30^ In the current study, we aimed to further understand which projection neurons bear this pathology. Additionally, we observed some larger inclusions in the putamen and aimed to understand if these were somatic Lewy bodies. The chosen subset of Lewy body cases displayed varying levels of Lewy pathology in the putamen, cingulate and frontal cortex in contrast to controls, which had no detectable pS129 α-synuclein (pSyn) pathology above the limit of detection (Fig. 2A and B). As reported in other studies,^13,39^ pathology in the cingulate cortex is more severe than in the frontal cortex. Almost all PD, PDD and DLB cases showed some degree of tau pathology, with the majority of PDD and DLB cases reaching Braak stage III–VI (Supplementary Fig. S1). To gain more quantitative insight into co-pathologies, we plotted quantitative tau and Aβ pathology by cases (Supplementary Fig. S2). Tau pathology was not elevated in the putamen, but showed increasing density in PD, PDD and DLB cortex (Supplementary Fig. S2A). Aβ pathology was increased across putamen and cortex, especially in DLB (Supplementary Fig. S2B).

**Figure 2:**
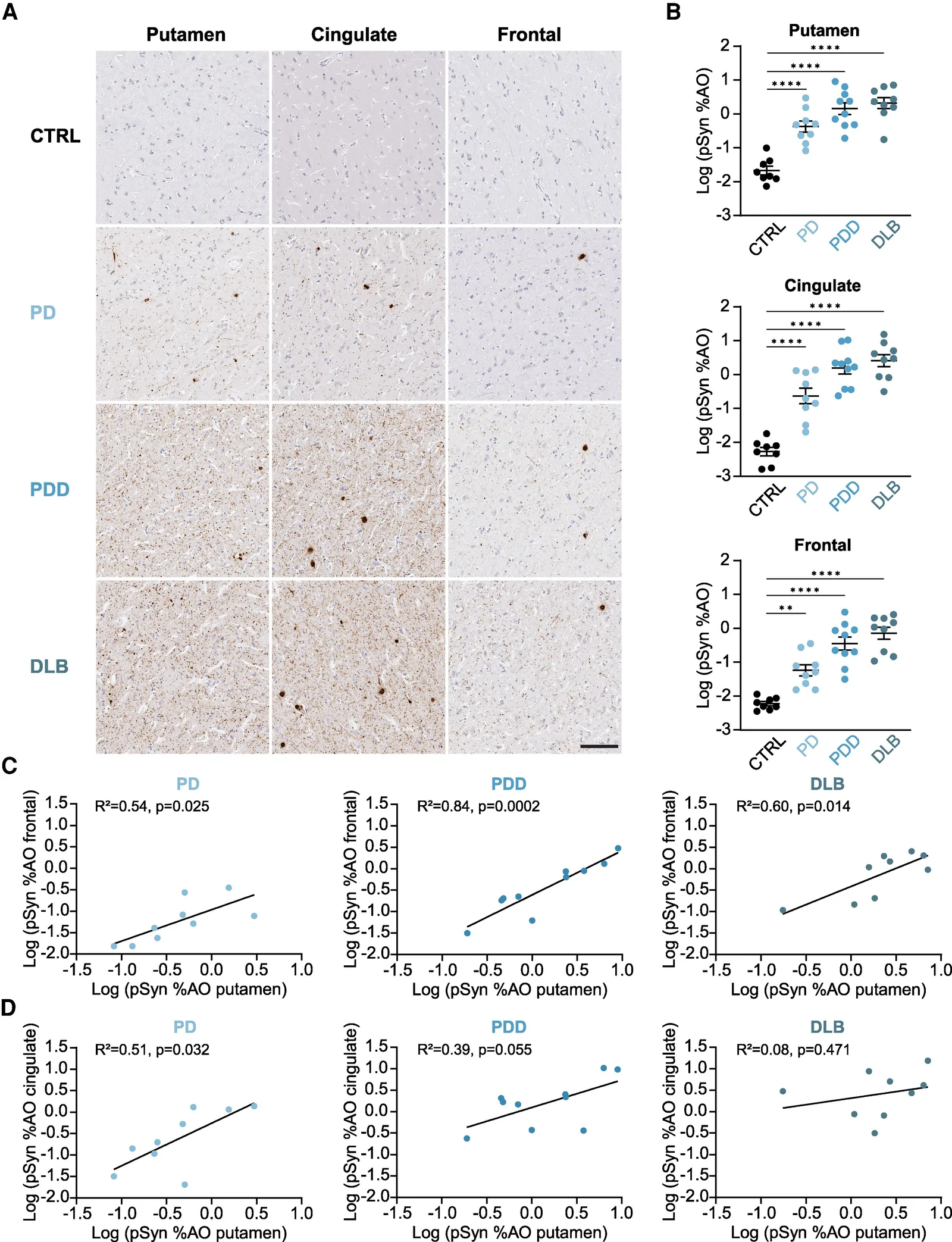
**Lewy pathology and neuropathological correlations in α-synucleinopathies.** (**A**) Representative staining of pS129 α-synuclein (pSyn) in the putamen, cingulate and frontal cortex of control (CTRL), Parkinson’s disease (PD), PD dementia (PDD) and dementia with Lewy bodies (DLB) cases. Scale bar = 100 μm. (**B**) Quantification of pSyn levels in putamen (*F*(3,32) = 29.41, *P* < 0.0001), cingulate (*F*(3,32) = 40.69, *P* < 0.0001) and frontal (*F*(3,32) = 29.82, *P* < 0.0001) cortex. Error bars represent mean ± SEM with individual cases plotted as dots. One-way ANOVA; Tukey’s multiple comparisons test. *P < 0.05, **P < 0.01, ***P < 0.001, ****P < 0.0001. (**C**) Scatter plots showing the correlation between pSyn levels in the putamen and frontal cortex of individual cases with PD (*F*(1,7) = 8.11, *P* = 0.0248), PDD (*F*(1,8) = 41.43, *P* = 0.0002) and DLB (*F*(1,7) = 10.47, *P* = 0.0143). (**D**) Scatter plots showing the correlation between pSyn levels in the putamen and cingulate cortex of individual donors with PD (*F*(1,7) = 7.19, *P* = 0.0315), PDD (*F*(1,8) = 5.07, *P* = 0.0545) and DLB (*F*(1,7) = 0.58, *P* = 0.4711). Lines represent the linear regression line of best fit. (**B–D**) Each dot represents data from individual donors. CTRL *n* = 8, PD *n* = 9, PDD *n* = 10, DLB *n* = 9. AO = area occupied.

Since one of the major inputs to the striatum is corticostriatal projections, we next investigated whether the burden of Lewy pathology in the putamen was related to cortical pathology. Lewy pathology levels in the frontal cortex were strongly correlated with those in the putamen across all groups (Fig. 2C). In contrast, pSyn levels in the cingulate showed a positive correlation with the putamen only in the PD group, with no significant correlation observed in the PDD or DLB groups (Fig. 2D). This lack of significance may be due to the overall high pathology burden in dementia cases, as across all cases, putamen pathology was correlated with cingulate pathology (Supplementary Fig. S3). Together, these findings suggest that the neuritic pathology in the putamen could represent corticostriatal projections.

### Tyrosine hydroxylase projections degenerate across LBDs and colocalize minimally with pSyn pathology

Dopaminergic neurons from the substantia nigra project to the putamen and are severely impacted in LBDs. Based on staining, these vulnerable dopaminergic neurons appear to undergo axonal dieback, and eventually, neurons are lost.^13,18,40,41^ Therefore, we first analysed the expression of dopaminergic terminals in the putamen using immunofluorescence labelling for TH (Fig. 3A). The putamen showed severe denervation in LBD cases (Fig. 3A and B), similar to previous reports.^18,27^ No significant differences were observed between α-synucleinopathies, suggesting that all patients were at an advanced stage of the disease, resulting in nearly complete striatal dopaminergic denervation at the time of death. While phenotypic downregulation of TH has been well-documented, especially with regards to counting dopaminergic neurons, previous work shows that the density of dopaminergic terminals in PD is nearly equivalent whether assessed by TH or DAT,^18^ and a recent study further confirmed that DAT terminals are nearly absent in PD cases,^34^ similar to our study. However, TH staining provides the advantage of detecting axonal α-synuclein inclusions in dopaminergic neurons even if they are not in presynaptic terminals.

**Figure 3:**
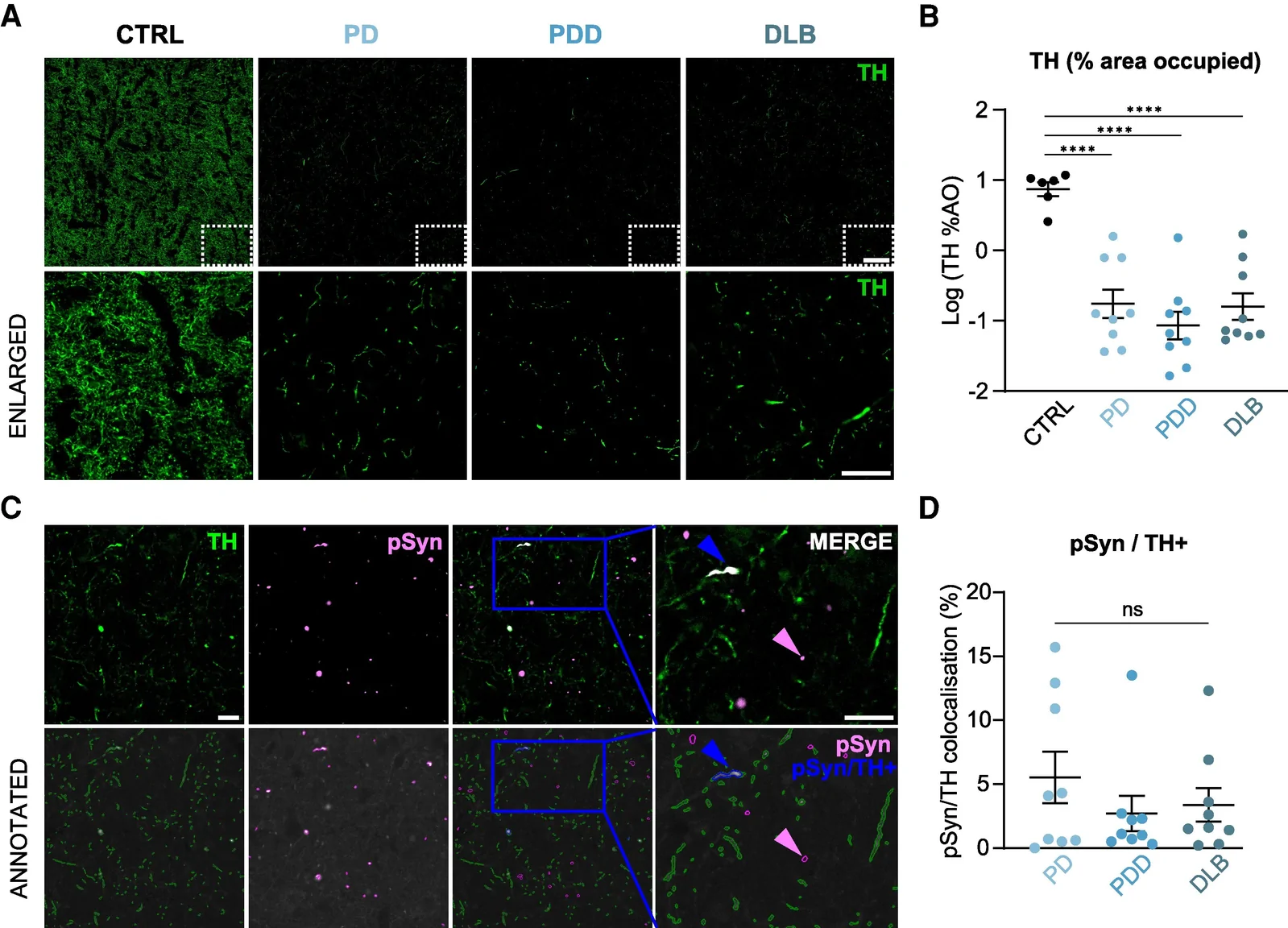
**Tyrosine hydroxylase projections degenerate across LBDs and colocalize minimally with pSyn pathology.** (**A**) Representative images of tyrosine hydroxylase (TH) immunofluorescence in the putamen of control (CTRL), Parkinson’s disease (PD), PD dementia (PDD) and dementia with Lewy bodies (DLB) cases. Scale bar = 50 μm (first row), 20 μm (second row; enlarged images). (**B**) Quantification of the percentage of TH-positive area across all groups (*F*(3,29) = 17.31, *P* < 0.0001). One-way ANOVA; Tukey’s multiple comparisons test. ****P < 0.0001. (**C**) Representative images of pSyn colocalization with TH fibres in the putamen (first row). Annotated images of object-based colocalization (second row): pSyn/TH+ (blue arrow) and pSyn not colocalized with TH (magenta arrow). Scale bars = 20 μm. (**D**) Quantification of the percentage of pSyn objects colocalizing with TH across all groups (*F*(2,15.58) = 0.65, *P* = 0.5376). Welch’s one-way ANOVA; Dunnett’s T3 multiple comparisons test. ns = non-significant. (**B and D**) Error bars represent mean ± SEM. Each dot represents data from individual donors. *n* = 6 for CTRL, *n* = 9 for PD, PDD, DLB. AO = area occupied.

To determine whether striatal Lewy pathology was present in the remaining TH fibres, we used an object-based colocalization approach to determine the portion of pSyn objects within TH-positive fibres. Confocal images co-stained for pSyn and TH were analysed using CellProfiler software, where pSyn aggregates colocalizing with TH-positive fibres were identified as pSyn/TH^+^ objects (Fig. 3C). Only a small fraction (2–6%) of pSyn aggregates colocalized with TH-positive fibres, with no significant differences observed across groups (Fig. 3D). Overall, these results suggest that, in our cohort, by the late stages of α-synucleinopathies, dopaminergic terminals in the putamen are already largely degenerated, and pSyn pathology accumulates predominantly outside the remaining dopaminergic fibres.

### Striatal Lewy pathology is enriched in VGLUT1 presynaptic terminals

The putamen receives dense excitatory input from the cerebral cortex, particularly from motor and associative regions. Disruption of this corticostriatal connectivity, driven by α-synuclein pathology and synaptic dysfunction, may contribute to cognitive decline and dementia.^17^ The putamen is predominantly composed of GABAergic neurons, and these neurons send collateral inputs within the putamen.^25,26^ To assess potential alterations in excitatory and inhibitory synaptic inputs, we quantified glutamatergic cortical and GABAergic terminals using immunofluorescence labelling for VGLUT1 and VGAT, respectively, followed by object-based analysis (Fig. 4A). No significant differences were observed in the VGLUT1 and VGAT puncta among groups (Fig. 4B and C). The number of pSyn objects was elevated in disease groups and was significantly higher in DLB compared to controls and PD (Fig. 4D).

**Figure 4:**
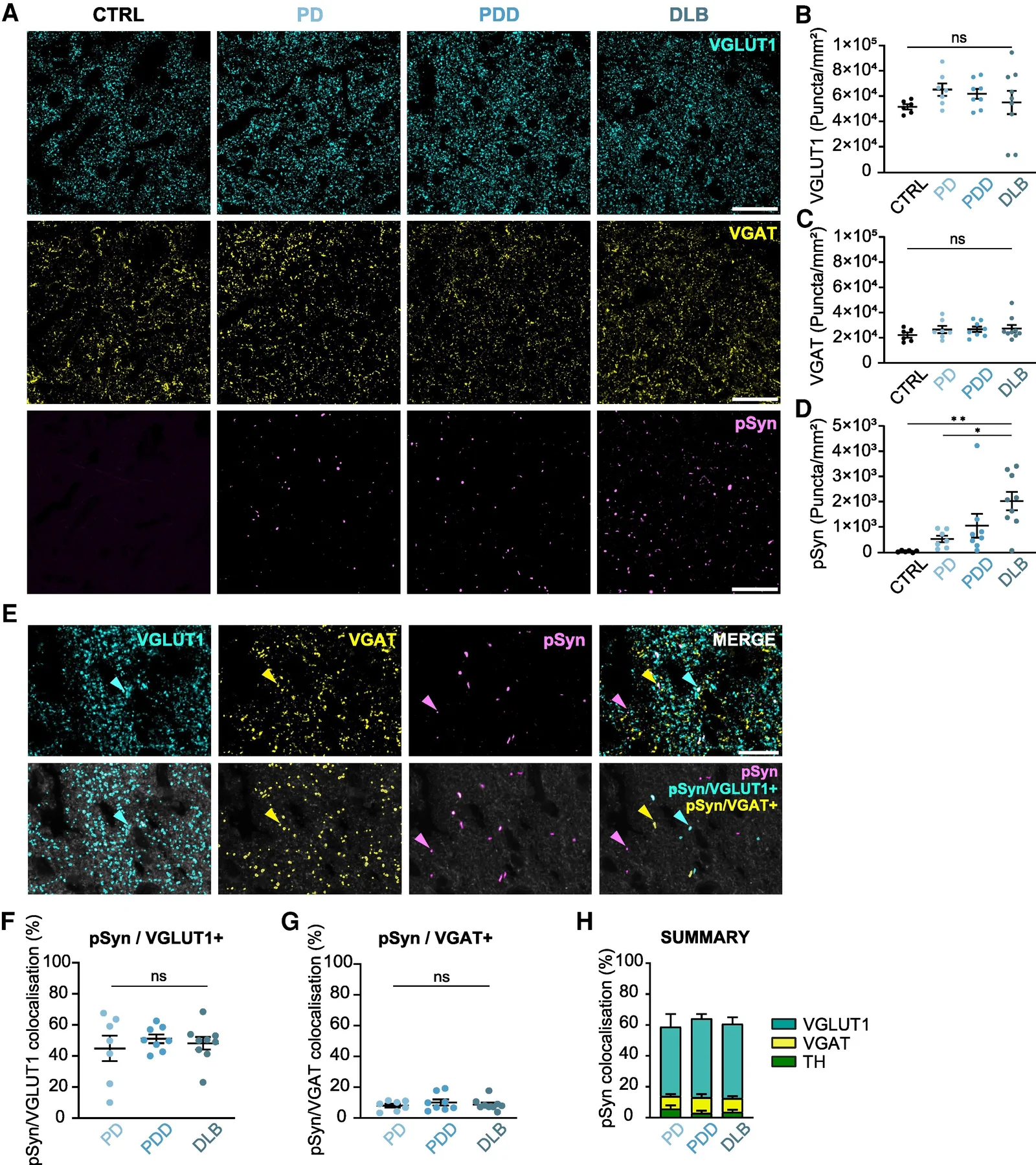
**Striatal Lewy pathology is enriched in VGLUT1 presynaptic terminals.** (**A**) Representative images of VGLUT1, VGAT and pSyn stainings in the putamen of control (CTRL), Parkinson’s disease (PD), PD dementia (PDD) and dementia with Lewy bodies (DLB) cases. Scale bar = 50 μm. (**B–D**) Quantification of VGLUT1 (*F*(3,26) = 0.85, *P* = 0.478) (**B**), VGAT (*F*(3,26) = 0.69, *P* = 0.565) (**C**) and pSyn (*F*(3,26) = 6.37, *P* = 0.002) (**D)** puncta per mm^2^ across groups. (**E**) Representative images of pSyn colocalization with presynaptic markers VGLUT1 and VGAT (top row) and annotated object-based colocalization (bottom row) from a DLB case: pSyn/VGLUT1^+^ (cyan arrowhead), pSyn/VGAT^+^ (yellow arrowhead) and pSyn without colocalization (magenta arrowhead). Scale bar = 20 μm. (**F–G**) Quantification of the percentage of pSyn objects colocalized with VGLUT1 (*F*(2,21) = 0.34, *P* = 0.716) (**F**) and VGAT (*F*(2,21) = 0.40, *P* = 0.675) (**G**) across groups. (**H**) pSyn colocalization expressed as a cumulative percentage in stacked bar format. (**B–D and F–H**) Error bars represent mean ± SEM, with each dot representing data from individual donors. *n* = 6 for CTRL, *n* = 7 for PD, *n* = 8 for PDD and *n* = 9 for DLB groups. One-way ANOVA; Tukey’s multiple comparisons test. *P < 0.05, **P < 0.01, ns = non-significant.

Using the same object-based colocalization approach applied in the TH analysis, we next quantified the proportion of pSyn aggregates colocalized with VGLUT1-positive or VGAT-positive synaptic terminals, defined as pSyn^+^/VGLUT1^+^ and pSyn^+^/VGAT^+^, respectively (Fig. 4E). We observed that pSyn aggregates mostly (∼50%) colocalized with VGLUT1-positive terminals (Fig. 4F). In contrast, colocalization with VGAT-positive terminals was minimal (∼9%) (Fig. 4G). Despite substantial Lewy pathology in the frontal and cingulate cortex, the pathology load did not correlate with the pSyn^+^/VGLUT1^+^ terminals (Supplementary Fig. S4). Collectively, these findings indicate that approximately half of Lewy pathology in the putamen is localized to glutamatergic presynaptic terminals; however, this enrichment is not accompanied by a reduction of VGLUT1-positive terminals.

### VGLUT1 presynaptic terminals containing pSyn pathology are enlarged

Many of the α-synuclein inclusions in the putamen were large enough to be small Lewy bodies. However, upon co-staining, these larger inclusions colocalized with larger VGLUT1-positive puncta (Fig. 5A). Recent studies have shown that Lewy pathology enrichment at presynaptic terminals may contribute to their swelling, dysfunction and eventual loss.^34,42^ To assess potential changes in synaptic morphology, we analysed the size of VGLUT1-positive presynaptic terminals with (VGLUT1/pSyn+) and without (VGLUT1) pSyn pathology using CellProfiler software (Fig. 5A). In the segmented images (second row), we observed enlarged VGLUT1 synapses colocalizing with pSyn, highlighted by orange arrows (Fig. 5A). We observed a 2-fold increase in the size of VGLUT1 presynaptic terminals containing pSyn compared to those without the pathology, but no significant differences were observed between LBD groups (Fig. 5B). These results demonstrate that while α-synuclein inclusions in the putamen are associated with dopaminergic terminal loss, glutamatergic terminals are preserved but exhibit dystrophic changes, potentially preceding subsequent synapse and neuron loss.

**Figure 5:**
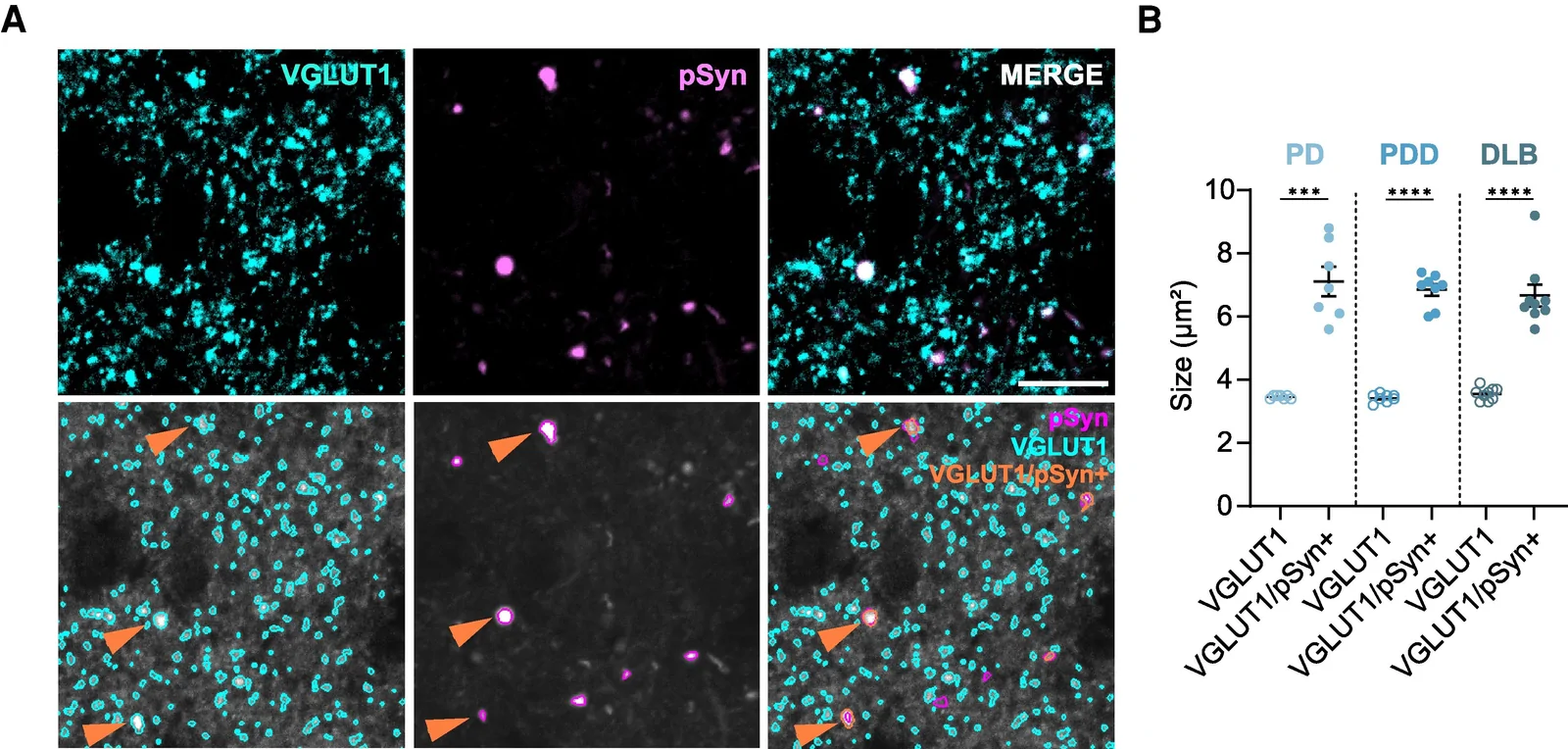
**Enlarged VGLUT1 terminals contain Lewy pathology.** (**A**) Representative images of pSyn colocalization with enlarged presynaptic marker VGLUT1 in the putamen (first row). Annotated images: VGLUT1 marker (cyan) and pSyn (magenta), enlarged VGLUT1/pSyn + (orange arrowhead), scale bar = 20 μm. (**B**) Quantification of the size of presynaptic markers VGLUT1 with (VGLUT1/pSyn+) or without (VGLUT1) colocalization of pSyn in Parkinson’s disease (PD), PD dementia (PDD) and dementia with Lewy bodies (DLB) cases. Error bars represent mean ± SEM, with each dot representing data from individual donors. Welch’s unpaired *t*-test for PD (*t*(6.02) = 8.28, *P* = 0.0002), PDD (*t*(7.84) = 18.06, *P* < 0.0001) and DLB (*t*(8.60) = 8.82, *P* < 0.0001) groups.

## Discussion

In this study, we aimed to identify which neurons projecting to the putamen bear neuritic Lewy pathology across LBDs and to determine how that pathology relates to structural changes in synapses. We found that (i) dopaminergic denervation was advanced across all disease groups, minimizing the extent of Lewy pathology in those terminals, (ii) Lewy pathology was selectively enriched in putamen glutamatergic VGLUT1-positive synaptic terminals, (iii) Lewy pathology was associated with enlargement of terminals but not overall presynaptic terminal loss in the putamen, and (iv) Lewy pathology in the putamen correlated with Lewy pathology in cortical regions. Taken together, our findings highlight a circuit-specific vulnerability of corticostriatal synapses and suggest that presynaptic Lewy pathology may contribute to cortical disease progression and cognitive impairment in LBDs.

α-Synuclein is a presynaptic protein involved in synaptic vesicle trafficking and neurotransmitter release, which plays a central role in the pathogenesis of LBDs.^31,43,44^ Misfolded α-synuclein can propagate trans-synaptically in model systems, spreading from cell-to-cell along anatomically and functionally connected networks.^45,46^ This prion-like property enables α-synuclein to propagate through vulnerable brain circuits, driving progressive synaptic dysfunction, neuronal death and disconnection within and between specific cell populations.^45,47^ However, despite evidence implicating α-synuclein in synaptic pathology, the enrichment of pSyn in specific cell-type terminals remains largely unexplored. Our data confirm and extend prior observations^13^ of a rostro-caudal gradient of Lewy pathology burden, showing significant pSyn accumulation in the putamen across PD, PDD and DLB, with progressively higher levels in the cingulate and frontal cortices paralleling the emergence and severity of cognitive symptoms.

The degeneration of nigrostriatal dopaminergic projections is a defining pathological feature of PD and related α-synucleinopathies. Clinically, it is estimated that by the time of PD diagnosis, 50–70% of striatal dopaminergic terminals have already been lost.^16,40,48^ This early and severe denervation is closely linked to the emergence of motor symptoms. In line with these observations, we observe a pronounced loss of TH-immunoreactive fibres in the putamen across all disease groups. The absence of significant differences among PD, PDD and DLB groups suggests that all cases were at an advanced disease stage, abrogating any differences in pathology distribution and denervation that may have existed earlier in the disease. As expected, we find that only a small proportion of pSyn aggregates colocalized with residual TH-positive fibres in the putamen, and this proportion is comparably low across all diagnostic groups. Our results are consistent with a recent study by Frigerio and colleagues that shows pSyn enrichment at DAT-positive terminals in incidental Lewy body disease, but not in PD.^34^ By examining across control, incidental Lewy body disease and PD groups, the study demonstrated that pSyn presence in dopaminergic synapses negatively correlates with the number of terminals, supporting a relationship between α-synuclein pathology and dopaminergic terminal loss. Consistent with our study, all PD cases had few dopaminergic terminals remaining, and pathology was largely in glutamatergic terminals in those cases. These results suggest that Lewy pathology shifts from dopaminergic to glutamatergic terminals in the putamen as the disease progresses.

α-Synuclein is enriched in excitatory synapses^49,50^ and Lewy pathology localizes to layer five intratelencephalic neurons in the postmortem PD cortex,^51^ highlighting glutamatergic neurons as one of the neuron types vulnerable to developing Lewy pathology. However, the extent to which pathological α-synuclein accumulates in corticostriatal synapses across LBD is only recently being explored. A key finding of this study is that approximately half of all pSyn aggregates in the putamen colocalize with VGLUT1-positive glutamatergic terminals across PD, PDD and DLB, while colocalization with VGAT-positive GABAergic terminals was consistently minimal. This selective synaptic enrichment supports a model wherein excitatory corticostriatal projections are a major site of α-synuclein aggregation, potentially reflecting local uptake of misfolded α-synuclein by cortical axon terminals within the striatum. Such a mechanism would be consistent with trans-synaptic propagation models, in which α-synuclein released by degenerating dopaminergic terminals or other affected neurons in the striatum is internalized by corticostriatal axons and retrogradely transported to cortical regions, contributing to the spread of pathology and cortical dysfunction in LBD. Interestingly, the proportion of VGLUT1-positive pSyn aggregates did not differ significantly across disease groups (∼50%), which may reflect a plateau effect in late-stage disease. The origin of the remaining pSyn signal that does not colocalize with either VGLUT1- or VGAT-positive terminals remains to be determined. One plausible interpretation is that these aggregates reside within excitatory axons that lack a presynaptic specialization in the imaging volume. Alternatively, a subset of pSyn aggregates may originate from neuronal populations not assessed in this study, such as thalamostriatal or brainstem-derived inputs. However, considering the established presynaptic enrichment of α-synuclein and its preferential association with excitatory terminals, it is likely that a significant proportion of these non-colocalized aggregates represent glutamatergic terminals lacking detectable synaptic markers.

We observed that VGLUT1-positive presynaptic terminals colocalizing with pSyn aggregates were significantly enlarged, suggesting both functional and morphological disruption. Indeed, neuritic α-synuclein aggregates have been shown to impair synaptic vesicle trafficking, resulting in vesicle clustering, synaptic swelling and eventually synapse pruning or loss.^33,52^ This is consistent with previous studies reporting that synaptic swelling and dysfunction are early events in PD, DLB and PD models in response to pSyn aggregates.^34,35,42^ Synaptic terminal enlargement in the striatum could reflect several non-mutually exclusive processes. One possibility is that non-degraded aggregates physically enlarge the presynaptic terminal.^53^ A second possibility is cytoskeletal and trafficking dysfunction: α-synuclein pathology has been linked to disruption of actin/spectrin and microtubule-dependent processes,^54-57^ which could impair vesicle recycling, axonal transport, and autophagic clearance, thereby causing local accumulation of membranous cargo and terminal swelling. This interpretation is conceptually similar to the dystrophic neurites observed around Aβ plaques, where transport and autophagic defects are associated with focal neuritic enlargement.^58^ A third possibility is compensatory hypertrophy of surviving synapses, as enlargement of remaining synaptic contacts has been described in neurodegenerative settings with synapse loss and may help preserve local synaptic strength.^59,60^ These mechanisms are not mutually exclusive and may coexist within the same striatal regions.

Corticostriatal glutamatergic transmission plays a critical role in the manifestation of motor and cognitive symptoms observed in LBD, and previous work supports synapses as particularly vulnerable compartments of neurons.^61-63^ Imaging with a PET ligand that binds to presynaptic protein SV2A has shown that people with PD have broad disruption of presynaptic terminals,^64^ including the localized loss of SV2A signal in the putamen.^65,66^ However, because SV2A is a broad presynaptic marker, its loss in the putamen may reflect the loss of dopaminergic terminals. To gain insight into glutamatergic terminals more specifically, a recent study used immunofluorescence labelling and synaptic density quantification and found no changes in VGLUT1 expression in incidental PD or across PD disease stages (Braak 1–6) in the striatum.^34^ Additionally, a semi-quantitative analysis by western blot and immunoautoradiography showed an increase in VGLUT1 in the putamen associated with a decrease in VGLUT1 expression level in frontal and temporal cortices.^67^ In agreement with the two human studies, we observe a non-significant trend towards increased VGLUT1- and VGAT-positive puncta in the putamen in PD and PDD compared to controls, suggesting that the overall abundance of cortical and inhibitory inputs to the putamen remains preserved at the terminal level despite clinically associated dementia symptoms. This contrast between preserved glutamatergic terminals and depleted dopaminergic terminals further supports the concept that relatively resilient neuronal populations accumulate α-synuclein pathology, whereas the more vulnerable populations are lost and therefore underrepresented in late stages of the disease. Nevertheless, variation in the level of VGLUT1 expression within the DLB group demonstrates that some cases may have substantial excitatory terminal loss. PDD and DLB brains harbour more amyloid-β plaques in the striatum than PD, and striatal plaques are associated with dementia severity.^68,69^ These observations raise the possibility that in DLB, compared to PD and PDD, striatal VGLUT1 loss could be driven in part by amyloid-β pathology.

This study has several limitations. First, our analysis focused primarily on VGLUT1-positive glutamatergic terminals, which selectively label cortical input to the striatum. As a result, we did not assess thalamostriatal projections, which are predominantly VGLUT2-positive.^70,71^ This was due to poor performance of the VGLUT2 antibody in human tissue. Therefore, we cannot exclude the possibility that a substantial fraction of pSyn aggregates may also localize to VGLUT2 terminals, particularly given that Lewy pathology is found in the thalamus of people with PD.^72^ Second, while we did not observe any changes in the density of VGLUT1-positive terminals in PD and PDD, this apparent resilience may reflect compensatory sprouting or an increase in structurally intact but functionally impaired synapses. Because our approach did not include assessment of postsynaptic specializations or synaptic function, we cannot determine whether these terminals are functionally competent or merely structurally retained. Third, we focused on pS129 α-synuclein due to its high specificity and sensitivity for pathological α-synuclein, but future work on other α-synuclein post-translational modifications, or conformations (conformation-selective antibodies or proximity ligation assays)^73-75^ may unveil further details about which forms of α-synuclein localize near synapses. Finally, our cases represent advanced stages of disease, although our findings are consistent with a prior study that assessed a broader range of Braak stages.^34^

Together, these findings support a model of selective vulnerability within corticostriatal circuits, where early synaptic α-synuclein aggregation may contribute to cortical disease progression and cognitive impairment in LBD.

## Supplementary Material

fcag409_Supplementary_Data

## Data Availability

The datasets used and/or analysed during the current study are available from the corresponding author upon reasonable request.
