# Supplementary material for: Lewy pathology accumulates in enlarged corticostriatal synapses in α-synucleinopathies
Source: Brain Commun. 2026 Oct 25;8(6):fcag409. doi: 10.1093/braincomms/fcag409 (PMC13739184; doi:10.1093/braincomms/fcag409)
Supplement: Supplementary file 1 [file fcag409_supplementary_data.docx]

**Supplementary Material for:**

**Lewy pathology accumulates in enlarged corticostriatal synapses in α-synucleinopathies**

Alice Prigent^1^, Aastha Singh^1^, Michael X. Henderson^1^

^1^Department of Neurodegenerative Science, Van Andel Institute, Grand Rapids, MI 49503, USA

Correspondence to:

Michael X. Henderson

michael.henderson@vai.org

| **Group** | **Sex** | **PMI** | **Age at death** | **Disease duration** | **Braak stage (I-III)** |
| --- | --- | --- | --- | --- | --- |
| **Control** | Female | 21 | 63 | N/A | 0 |
| **Control** | Male | 8.5 | 62 | N/A | I |
| **Control** | Male | 24 | 57 | N/A | I |
| **Control** | Male | 17 | 59 | N/A | I |
| **Control** | Female | 21 | 56 | N/A | 0 |
| **Control** | Male | 16 | 62 | N/A | I |
| **Control** | Male | 13 | 71 | N/A | 0 |
| **Control** | Male | 20 | 66 | NA | 0 |
| **PD** | Female | 21 | 80 | 11 | I |
| **PD** | Female | 12 | 76 | N/A | I |
| **PD** | Male | 16 | 86 | 26 | I |
| **PD** | Male | 21.5 | 58 | 8 | I |
| **PD** | Female | 3.5 | 77 | 11 | I |
| **PD** | Male | 3.5 | 62 | 9 | I |
| **PD** | Male | 7 | 84 | 19 | II |
| **PD** | Female | 17 | 79 | 20 | III |
| **PD** | Female | 5.5 | 72 | 22 | I |
| **PDD** | Male | 7.5 | 78 | 25 | I |
| **PDD** | Male | 4 | 79 | 9 | I |
| **PDD** | Male | 4 | 64 | 17 | I |
| **PDD** | Male | 5 | 73 | 14 | 0 |
| **PDD** | Male | 20 | 63 | 23 | I |
| **PDD** | Male | 5 | 71 | 7 | II |
| **PDD** | Male | 10 | 59 | 20 | 0 |
| **PDD** | Female | 13 | 64 | 21 | I |
| **PDD** | Male | 4 | 68 | 8 | I |
| **PDD** | Male | 13 | 91 | 8 | II |
| **DLB** | Male | 24 | 71 | 9 | II |
| **DLB** | Male | 30 | 77 | 8 | III |
| **DLB** | Male | 16 | 80 | 9 | II |
| **DLB** | Male | 9 | 71 | 9 | III |
| **DLB** | Male | 12 | 68 | 8 | I |
| **DLB** | Male | 17 | 62 | N/A | I |
| **DLB** | Male | 6 | 70 | 6 | I |
| **DLB** | Female | 6 | 69 | 5 | III |
| **DLB** | Male | 21 | 67 | 5 | II |

**Table S1. Patients’ demographics.**

PMI: postmortem interval. Braak stages for the cohort are represented as I (combined stages I and II), II (combined stages III and IV) and III (combined stages V and VI).

**Figure S1. Braak stage correlations in α-synucleinopathies**. **A.** The percentages of cases assigned Braak stages 0, I-II, III-IV and V-VI is indicated for control (CTRL), Parkinson’s disease (PD), Parkinson’s disease with dementia (PDD) and dementia with Lewy bodies (DLB) cases. **B.** Cumulative scatter plot showing the correlation between Braak stage and pSyn levels in the putamen (F_1,2_ = 0.3272, p = 0.6250), cingulate cortex (F_1,2_ = 0.4364, p = 0.5768) and frontal cortex (F_1,2_ = 4.684, p = 0.1629) for all α-synucleinopathy cases. Line on each plot represents the linear regression line of best fit. Each dot represents data from individual donors. CTRL *n* = 8, PD *n =* 9, PDD *n* = 10, DLB *n* = 9.

**Figure S2. Co-pathologies in α-synucleinopathy study cohort**. **A.** Quantification of phosphorylated Tau (pTau) levels in putamen (F_3,32_ = 1.72, p = 0.182), cingulate (F_3,32_ = 12.52, p < 0.0001) and frontal (F_3,32_ = 3.47, p = 0.027) cortex. **B.** Quantification of amyloid-β (Aβ) levels in putamen (F_3,32_ = 2.83, p = 0.054), cingulate (F_3,32_ = 4.50, p = 0.0096) and frontal (F_3,32_ = 3.72, p = 0.021) cortex. Error bars represent mean ± S.E.M. with individual donors plotted as dots. Control (CTRL) *n* = 8, Parkinson’s disease (PD) *n =* 9, Parkinson’s disease with dementia (PDD) *n* = 10, dementia with Lewy bodies (DLB) *n* = 9. One-way ANOVA; Tukey’s multiple comparisons test. *p < 0.05, **p < 0.01, ****p < 0.0001.

**Figure S3. Neuropathological correlations in α-synucleinopathies**. **A.** Scatter plot showing the correlation between pSyn levels in the putamen and cingulate cortex (F_1,26_ = 23.76, p < 0.0001). **B.** Scatter plot showing the correlation between pSyn levels in the putamen and frontal cortex (F_1,26_ = 80.66, p < 0.0001). Line on each plot represents the linear regression line of best fit. Each dot represents data from individual donors. Parkinson’s disease (PD) *n =* 9, Parkinson’s disease with dementia (PDD) *n* = 10, dementia with Lewy bodies (DLB) *n* = 9.

**Figure S4.** Correlation of cortical pSyn pathology load with corticostriatal synapses with pathology in cases of Parkinson’s disease (PD, *n* =7; blue), Parkinson’s disease with dementia (PDD, *n* =8; green) and dementia with Lewy bodies (DLB, *n* =9; magenta). **A.** Scatter plot presenting correlation between the percentage of pSyn pathology colocalizing in VGLUT1+ terminals and pSyn pathology in the frontal cortex. **B.** Scatter plot presenting correlation between the percentage of pSyn pathology colocalizing in VGLUT1+ terminals and pSyn pathology in the cingulate cortex. Lines represent the linear regression line of best fit. Each data point represents individual donors.
